# Efficacy of neoadjuvant immunochemotherapy and survival surrogate analysis of neoadjuvant treatment in IB–IIIB lung squamous cell carcinoma

**DOI:** 10.1038/s41598-024-54371-8

**Published:** 2024-03-06

**Authors:** Jiacong Liu, Linhai Zhu, Muhu Tang, Xuhua Huang, Chen Gu, Cheng He, Xiayi Lv, Jian Hu

**Affiliations:** 1 Department of Thoracic Surgery, The First Affiliated Hospital, School of Medicine, Zhejiang University, No. 79 Qingchun Road, Hangzhou, 310003 China; 2 Key Laboratory of Clinical Evaluation Technology for Medical Device of Zhejiang Province, Hangzhou, 310003 China

**Keywords:** Immunochemotherapy, Chemotherapy, Neoadjuvant treatment, Lung squamous cell carcinoma (LUSC), Surgery, Cancer, Immunology, Oncology

## Abstract

Until now, there are still few comparisons between neoadjuvant immunochemotherapy and chemotherapy as first-line treatment for patients with stage IB-IIIB lung squamous cell carcinoma (LUSC). In addition, the ability of pathologic response to predict long-term survival has still not been established. In this retrospective, controlled clinical trial, we ultimately enrolled 231 patients with stage IB to IIIB LUSC who received 2–4 cycles perioperative immunochemotherapy or chemotherapy alone, followed by resection. The primary endpoint of this study was pathological response. Secondary endpoints were disease-free survival (DFS), overall survival (OS), objective response rate (ORR), surgical resection rate and adverse events (AEs). The rates of major pathologic response (MPR) and pathologic complete response (pCR) in the immunochemotherapy group were 66.7% and 41.9%, respectively, which were both higher than that in the other group (MPR: 25.0%, pCR: 20.8%) (P < 0.001). The median DFS in the chemotherapy group was 33.1 months (95% CI 8.4 to 57.8) and not reached in the immunochemotherapy group (hazard ratio [HR] for disease progression, disease recurrence, or death, 0.543; 95% CI 0.303 to 0.974; P = 0.038). The median OS of the immunochemotherapy group was not achieved (HR for death, 0.747; 95% CI 0.373 to 1.495; P = 0.41), with the chemotherapy group 64.8 months (95% CI not reached to not reached). The objective response rate (ORR) of immunochemotherapy regimen was higher than that of the chemotherapy regimen (immunochemotherapy: 74.5%, chemotherapy: 42.3%, P < 0.001). About 60.8% in the immunochemotherapy group and 61.5% in the chemotherapy group eventually underwent surgery. The incidence of grade3 and 4 adverse events was 18.3% in the immunochemotherapy group and 2.6% in the chemotherapy group. MPR was significantly associated with DFS and OS (HR, 0.325; 95% CI 0.127 to 0.833; P = 0.019; and HR, 0. 906; 95% CI 0.092 to 1.008; P = 0.051, respectively). The C-index of MPR (0.730 for DFS, 0.722 for OS) was higher than the C-index of cPR (0.672 for DFS, 0.659 for OS) and clinical response (0.426 for DFS, 0.542 for OS). Therapeutic regimen (P < 0.001; OR = 7.406; 95% CI 3.054 to 17.960) was significantly correlated with MPR. In patients with stage IB to IIIB LUSC, neoadjuvant treatment with immunochemotherapy can produce a higher percentage of patients with a MPR and longer survival than chemotherapy alone. MPR may serve as a surrogate endpoint of survival to evaluate neoadjuvant therapy.

## Introduction

Approximately 25–30% of lung cancers are pathologically classified as lung squamous cell carcinoma (LUSC)^1^. LUSC is primarily related to tobacco exposure and more commonly found in males^2^. Utilizing PD-L1/PD-1 immune checkpoint inhibitors, either alone or in combination with chemotherapy, proves highly suitable and effective for its treatment, aiming to minimize the need for reevaluation^3–6^. Findings from the KEYNOTE-407 and IMPOWER-131 studies demonstrate that employing immunochemotherapy as a first-line treatment can yield superior survival benefits compared to traditional chemotherapy for individuals diagnosed with stage IV LUSC^7,8^. Checkmate-816 study found that immunochemotherapy resulted in significantly longer event-free survival than chemotherapy in patients with stage IB-IIIA non–small-cell lung cancer (NSCLC) and NADIM II study demonstrated that immunochemotherapy produced longer survival than chemotherapy in patients with stage IIIA-IIIB NSCLC^9,10^. However, there are still few comparisons between neoadjuvant immunochemotherapy and chemotherapy as first-line treatment for patients with stage IB-IIIB LUSC.

Nowadays, overall survival (OS) is still the gold indicator for evaluating the endpoint of neoadjuvant therapy, but it requires a long period of follow-up and a large number of cases to obtain^11^. Therefore, many alternative indicators for OS have gradually emerged in clinical practice, such as objective response rate (ORR), progression-free survival (PFS), disease-free survival (DFS), event-free survival (EFS), molecular residual disease (MRD), circulating tumor DNA (ctDNA), pathologic complete response (pCR), major pathologic response (MPR) etc. In recent years, an increasing number of clinical studies have used MPR as endpoints for evaluating the efficacy of neoadjuvant therapy^12^. At present, relevant evidence suggests that MPR and pCR associated with longer OS^13,14^, but the ability of pathologic response to predict long-term survival has still not been established and the factors influencing pathologic response still failed to reach an agreement.

Therefore, in this study, we compare the safety and efficacy of neoadjuvant immunochemotherapy and chemotherapy as first-line treatment for patients with stage IB-IIIB LUSC. In addition, survival surrogate analysis of neoadjuvant therapy is performed in patients with stage IB-IIIB LUSC to evaluate the prognostic value of pathologic response and investigate the influencing factors of pathologic response.

## Methods

### Study design and patients

This retrospective, controlled clinical trial was carried out at the Department of Thoracic Surgery, the First Affiliated Hospital, Zhejiang University School of Medicine. Inclusion criteria were as follows: (I) age over 18 and under 80 years; (II) histopathologically diagnosed, previously untreated stage IB-IIIB LUSC (according to the eighth edition of the AJCC TNM staging^15^) that was deemed to be surgically resectable by a multidisciplinary clinical team; (III) Eastern Cooperative Oncology Group (ECOG) performance status of 0 or 1. Exclusion criteria were below: (I) absence of essential pre-treatment imaging evaluations in our hospital; (II) imaging examinations no more than 2 times; (III) previous anticancer treatment, including radiotherapy, interventional therapy or drug treatment; (IV) infectious or autoimmune disease; (V) current systemic immunosuppressive treatments; (VI) other concomitant malignant tumors; and (VII) distant metastases.

This study received approval from the Clinical Research Ethics Committee of the First Affiliated Hospital, Zhejiang University School of Medicine (2021 IIT No. 844), adhering to the principles outlined in the 2013 revised Declaration of Helsinki and the Good Clinical Practice Guidelines. Prior to participation, written informed consent was obtained from all patients.

### Treatment procedures

Included patients underwent 2–4 cycles of preoperative chemotherapy (3 weeks per cycle) between November 2015 and March 2022, or received immunochemotherapy from January 2019 to March 2022. The immunotherapy regimen included camrelizumab (200 mg), durvalumab (1000 mg), nivolumab (200 mg), sintilimab (200 mg), tislelizumab (200 mg), or pembrolizumab (200 mg). The chemotherapy regimen consisted of etoposide at 100 mg/m^2^, cisplatin at 75 mg/m^2^, or carboplatin with an AUC (area under the ROC curve representing drug plasma concentration) of 5. After two cycles of neoadjuvant treatment, patients would undergo evaluation to assess surgical suitability. In the event of patient intolerance to neoadjuvant therapy, we will either modify the treatment plan or contemplate postponing it. If there is no substantial tumor regression after 1–2 additional cycles of treatment, we will continue treatment and assess the surgical opportunity. In case of disease progression, we will recommend radiotherapy.

Prior to commencing neoadjuvant treatment, patients undergo a comprehensive series of imaging evaluations, encompassing chest and abdominal computed tomography (CT), endoscopic ultrasound, positron emission tomography (PET)–CT, bone emission computed tomography, brain magnetic resonance imaging, and ultrasound scans. These evaluations serve to assess the tumor status and establish baseline data. During neoadjuvant treatment, chest CT was done every 2 cycles until either surgery was conducted or the patient discontinued treatment. We conducted routine blood and biochemical examinations weekly, while assessments of myocardial enzyme spectrum, thyroid function, and coagulation function were performed every three weeks. Patients' evaluations were used to assess gastrointestinal and skin reactions. Surgical approaches encompassed open radical surgery, video-assisted thoracoscopic surgery (VATS), and robot-assisted thoracoscopic surgery (RATS), all of which included routine lymph node dissection. The scope of lymph node dissection comprised a minimum of three lung lymph node groups and three mediastinal lymph node groups, with mandatory inclusion of subcarinal lymph nodes. The dissection generally encompassed lymph nodes on the left side, spanning from Group 3 to 4L and from 5 to 13, as well as lymph nodes on the right side, covering Group 3a, 4R, and 7 to 13. After surgery, imaging assessments would be performed every 1–3 months. And adjuvant treatment (like immunochemotherapy, immunotherapy, chemotherapy or radiotherapy) would be considered. The follow-up period concluded either after a minimum of one year post-surgery, or when the patient chose to discontinue treatment, or upon the study's termination, in order to reduce the recheck rate.

### End points and assessments

The primary endpoint of this study was pathological response. Tumor regression grade (TRG) was adopted to express pathological response. Pathological complete remission (pCR) and major pathological response (MPR) are considered equivalent to TRG 0 and TRG 0–1, respectively, in accordance with the guidelines established by the College of American Pathologists (CAP) and The National Comprehensive Cancer Network (NCCN). In these guidelines, TRG 0 signifies the absence of viable tumor cells, TRG 1 denotes the presence of viable tumor cells at a rate of ≤ 10%, TRG 2 characterizes viable tumor cells within the range of 10% to ≤ 50%, and TRG 3 signifies the presence of viable tumor cells exceeding 50%.

Secondary endpoints were objective response rate (ORR), disease-free survival (DFS) and overall survival (OS). The tumor treatment response was assessed on the basis of the Response Evaluation Criteria in Solid Tumor version 1.1 (RECIST 1.1)^16^ complete response (CR): complete disappearance of all target lesions, partial remission (PR): ≥ 30% reduction in the total diameter of target lesions, progressive disease (PD): ≥ 20% increase in the total diameter of target lesions or the appearance of new lesions, stable disease (SD): neither CR, PR nor PD. Objective response rate (ORR) was composed of CR and PR. DFS was defined as the time from surgical resection to disease progression according to RECIST 1.1 or death, whichever occurred first. OS was defined as the time from surgical resection until death from any cause. Other secondary endpoints included surgical resection rate and adverse events (graded according to Common Terminology Criteria for Adverse Events [CTCAE] version 5.0^17^).

### Statistical analysis

Categorical variables were reported using frequencies and percentages, and group comparisons were performed using either the chi-square test or Fisher's exact test. Continuous variables were represented by the median and interquartile range (IQR), and group differences were assessed using the t-test or Wilcoxon test. For the evaluation of disease-free survival (DFS) and overall survival (OS) in the post-operative (PP) population, we employed the Kaplan–Meier method, with group comparisons accomplished through the stratified log-rank test. Median follow-up time was determined using the reverse Kaplan–Meier method. Cox proportional-hazards models were utilized to assess the association between each study variable and survival outcomes, aiming to minimize the need for rechecks. Harrell's concordance index (C-index) was computed to assess the survival surrogate's capability in distinguishing between deceased and surviving patients, as well as between progressing and non-progressing patients, with the goal of reducing the need for rechecks. Binary logistic regression model was used to screen the influencing factors of MPR. Analyses were performed with R software (version 4.1.2). A two-sided P value < 0.05 was considered to be statistically significant.

### Ethical statement

This trial was approved by the Clinical Research Ethics Committee of the First Affiliated Hospital, Zhejiang University School of Medicine (2021 IIT No. 844), and done in accordance with the Declaration of Helsinki (as revised in 2013) and Good Clinical Practice Guidelines. Written informed consent was obtained from patients so that we could acquire and use required information from their medical record in our hospital.

## Results

### Patients and treatments

**Figure 1:**
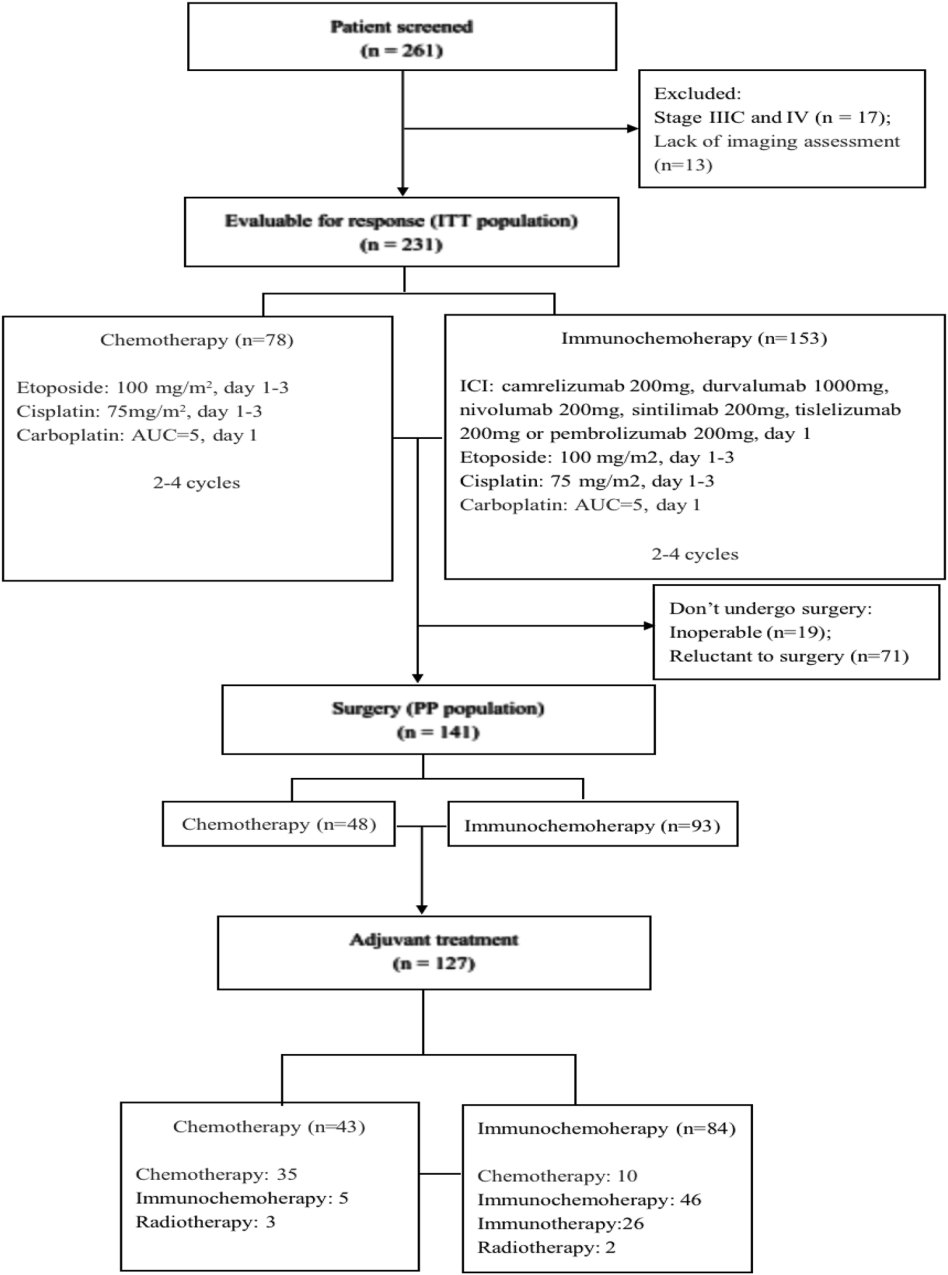
The flowchart of case screening. ITT, intention-to-treat; PP, postoperative.

**Figure 2:**
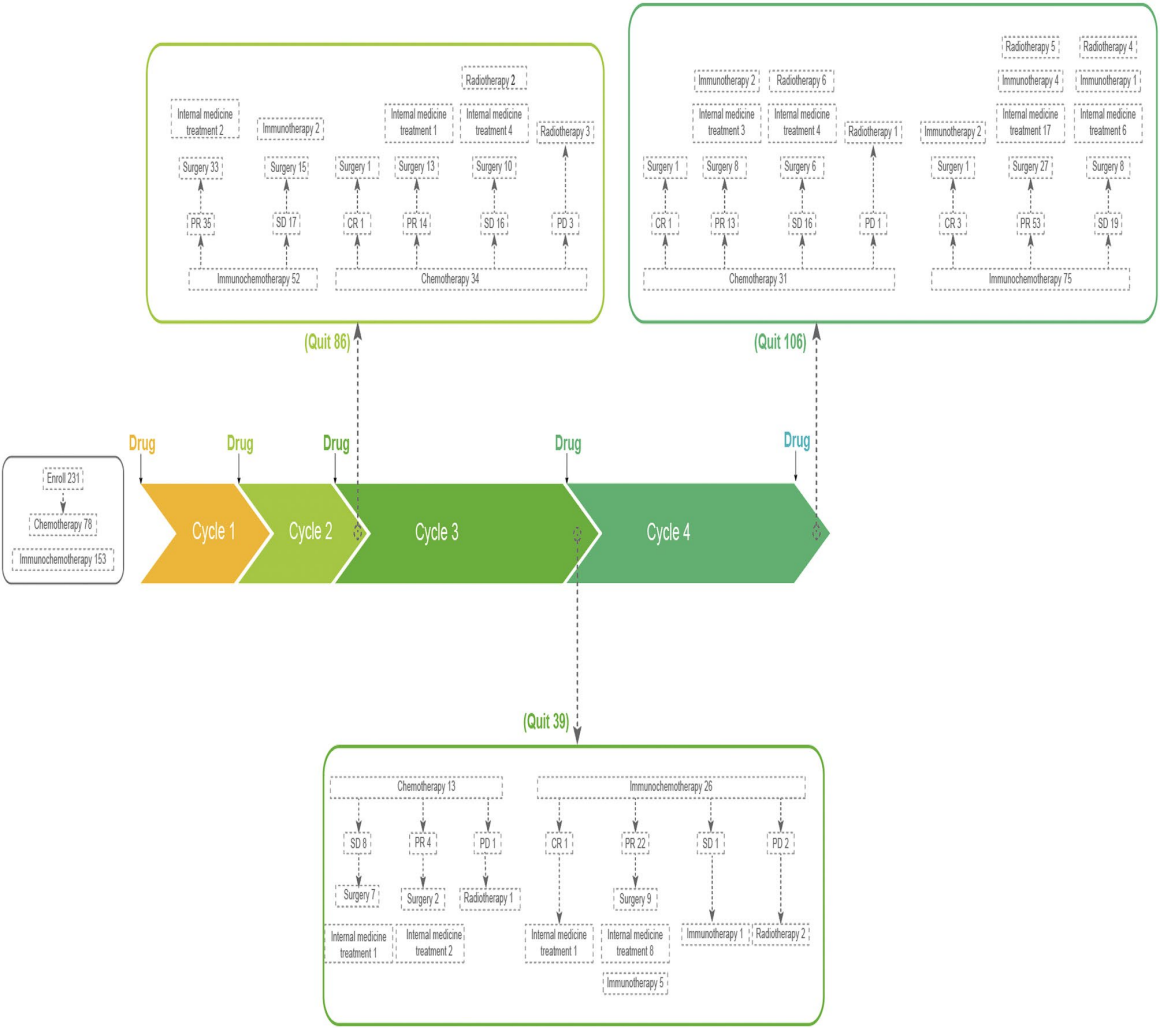
The overview of preoperative treatment process in the ITT population (n = 231). ITT, intention-to-treat; CR, complete remission; PR, partial remission; SD, stable disease; PD, progression disease. *Internal medicine treatment refers to no anticancer treatment in our hospital.

From November 2015 to March 2022, a total of 261 patients were screened, and 231 patients (ITT population) were consecutively enrolled to receive neoadjuvant immunochemotherapy (n = 153) or chemotherapy (n = 78) (Fig. 1). Characteristics of ITT population at baseline were shown in Table 1. There were no statistically significant differences between the two groups in terms of age, sex, ECOG performance status, smoking and drinking habits, comorbidities, tumor location, clinical stage, or treatment cycle, thus reducing the need for rechecks. The overview of preoperative treatment process in the ITT population was listed in Fig. 2. Among the ITT population, 141 patients (PP population) had surgery (immunochemotherapy group: 93, chemotherapy group: 48). Adjuvant treatment (such as: immunochemotherapy, immunotherapy, chemotherapy or radiotherapy) was received by 90.3% (84/93) of the patients in the immunochemotherapy group and 89.6% (43/48) of those in the chemotherapy group.

**Table 1:** Characteristics of the ITT population (n = 231) at baseline, according to neoadjuvant regimen.

Characteristic	Total, n = 231	Chemotherapy, n = 78	Immunochemotherapy, n = 153	*P*-value
Median age (IQR), years	65.7 (60.0–71.0)	63.1 (58.8–68.3)	67.1 (62.0–72.0)	0.402
Sex, n (%)				0.510
Male	226 (97.8)	77 (98.7)	149 (97.4)	
Female	5 (2.2)	1 (1.3)	4 (2.6)	
ECOG performance status, n (%)				0.490
0	156 (67.5)	55 (70.5)	101 (66.0)	
1	75 (32.5)	23 (29.5)	52 (34.0)	
Smoking status, n (%)				0.121
Never	62 (26.8)	16 (20.5)	46 (30.1)	
Ever	169 (73.2)	62 (79.5)	107 (69.9)	
Drinking status, n (%)				0.433
Never	153 (66.2)	49 (62.8)	104 (68.0)	
Ever	78 (33.8)	29 (37.2)	49 (32.0)	
Comorbidities, n (%)				
Pulmonary disease	32 (13.9)	8 (10.3)	24 (15.7)	0.463
Cardiac disease	18 (7.8)	5 (6.4)	13 (8.5)	0.576
Diabetes mellitus	23 (10.0)	11 (14.1)	12 (7.8)	0.133
Hypertension	66 (28.6)	22 (28.2)	44 (28.8)	0.930
Tumor location, n (%)				0.878
Mediastinum	3 (1.3)	1 (1.3)	2 (1.3)	
Hilum of left lung	6 (2.6)	1 (1.3)	5 (3.3)	
Hilum of right lung	2 (0.9)	1 (1.3)	1 (0.7)	
Inferior lobe of left lung	34 (14.7)	13 (16.7)	21 (13.7)	
Inferior lobe of right lung	46 (19.9)	18 (23.1)	28 (18.3)	
Middle lobe of right lung	14 (6.1)	3 (3.8)	11 (7.2)	
Superior lobe of left lung	71 (30.7)	24 (30.8)	47 (30.7)	
Superior lobe of right lung	55 (23.8)	17 (21.8)	38 (24.8)	
Clinical stage, n (%)				0.699
IB	5 (2.2)	1 (1.3)	4 (2.6)	
IIA	3 (1.3)	1 (1.3)	2 (1.3)	
IIB	26 (11.3)	9 (11.5)	17 (11.1)	
IIIA	107 (46.3)	32 (41.0)	75 (49.0)	
IIIB	90 (39.0)	35 (44.9)	55 (35.9)	
Treatment cycle, n (%)				0.324
2	86 (37.2)	34 (43.6)	52 (34.0)	
3	39 (16.9)	13 (16.7)	26 (17.0)	
4	106 (45.9)	31 (39.7)	75 (49.0)	
Immunotherapy regimes, n (%)				NA
Camrelizumab, 200 mg	49 (21.2)	0 (0.0)	49 (32.0)	
Durvalumab, 1000 mg	2 (0.9)	0 (0.0)	2 (1.3)	
Nivolumab, 200 mg	22 (9.5)	0 (0.0)	22 (14.4)	
Sintilimab, 200 mg	17 (7.4)	0 (0.0)	17 (11.1)	
Tislelizumab, 200 mg	32 (13.9)	0 (0.0)	32 (20.9)	
Pembrolizumab, 200 mg	31 (13.4)	0 (0.0)	31 (20.3)	

ITT, intention-to-treat; IQR, interquartile range; ECOG, Eastern Cooperative Oncology Group.

### Surgery

Among the ITT population, 60.8% (93/153) in the immunochemotherapy group and 61.5% (48/78) in the chemotherapy group eventually underwent surgery. Outcomes of surgery were summarized in Table 2. The time from first neoadjuvant treatment to surgery was longer in the immunochemotherapy group (P = 0.085). Open surgery was performed in most of patients of these two groups (immunochemotherapy group:62.4%, chemotherapy group:70.8%). The rate of conversion from video-assisted thoracoscopic surgery to open surgery in the immunochemotherapy group and chemotherapy group was 16.1% and 12.5%, respectively. Lobectomy and sleeve lobectomy were the main surgical methods in these two groups. In addition, 94.6% of the patients in the immunochemotherapy group and 77.8% of those in the chemotherapy group underwent R0 resection. As we could see from the ypTNM stage, there were more patients in stage 0 and stage IA in the immunochemotherapy group (P = 0.058). In general, the incidence of postoperative complications in the immunochemotherapy group was higher than that in the chemotherapy group. Hydrothorax (29.0% vs 12.5%, P = 0.028) and pneumothorax (7.1% vs 0.0%, P = 0.051) were more common in the immunochemotherapy group. The hydropneumothorax occurring rate was obviously lower in the immunochemotherapy group than that in the chemotherapy group (10.8% vs 27.1%, P = 0.013). There were no perioperative deaths in both groups.

**Table 2:** Surgical outcomes of the PP population (n = 141).

	Total, n = 141	Chemotherapy, n = 48	Immunochemotherapy, n = 93	*P*-value
Time from first treatment to surgery, median (IQR), day	79.2 (52.0–100.0)	75.4 (51.0–111.0)	81.2 (51.5–101.5)	0.085
Surgical approach, n (%)				0.527
Open	92 (65.2)	34 (70.8)	58 (62.4)	
VATS	25 (17.7)	8 (16.7)	17 (18.3)	
RATS	3 (2.1)	0 (0.0)	3 (3.2)	
VATS-open	21 (14.9)	6 (12.5)	15 (16.1)	
Operation method, n (%)				0.547
Wedge resection	3 (2.1)	1 (2.1)	2 (2.2)	
Segmental resection	2 (1.4)	0 (0.0)	2 (2.2)	
Lobectomy	74 (52.5)	30 (62.5)	44 (47.3)	
Sleeve lobectomy	42 (29.8)	11 (22.9)	31 (33.3)	
Exploratory thoracotomy	5 (3.5)	1 (2.1)	4 (4.3)	
Total pneumonectomy	15 (10.6)	5 (10.4)	10 (10.8)	
Operation time, median (IQR), min	165.6 (116.0–211.0)	169.6 (107.0–154.0)	163.6 (109.3–195.8)	0.581
Estimated blood loss, median (IQR), mL	102.7 (30.0–100.0)	115.9 (50.0–200.0)	96.1 (20.0–100.0)	0.880
Number of lymph node dissections during surgery, median (IQR), n	17.2 (10.0–21.5)	14.6 (9.0–18.0)	18.5 (11.0–24.0)	0.141
Surgical margin, n (%)				0.217
R0 resection	130 (92.2)	42 (87.5)	88 (94.6)	
R1 resection	8 (5.7)	5 (10.4)	3 (3.2)	
R2 resection	3 (2.1)	1 (2.1)	2 (2.2)	
Length of hospital stay, median (IQR), day	13.8 (9.0–17.8)	15.7 (11.0–19.0)	12.8 (8.0–16.0)	0.320
Postoperative complication, n (%)				
Overall complication	64 (45.4)	19 (39.6)	45 (48.4)	0.372
Hydropneumothorax	23 (16.3)	13 (27.1)	10 (10.8)	**0.013**
Hydrothorax	33 (23.4)	6 (12.5)	27 (29.0)	**0.028**
Pneumothorax	7 (5.0)	0 (0.0)	7 (7.1)	0.051
Chylothorax	1 (0.7)	0 (0.0)	1 (1.0)	0.471
Bronchial obstruction	1 (0.7)	0 (0.0)	1 (1.0)	0.471
ypTNM stage, n (%)				0.058
0	49 (34.8)	10 (20.8)	39 (41.9)	
IA	27 (19.1)	8 (16.7)	19 (20.4)	
IB	8 (5.7)	4 (8.3)	4 (4.3)	
IIA	3 (2.1)	1 (2.1)	2 (2.2)	
IIB	26 (18.4)	9 (18.8)	17 (18.3)	
IIIA	22 (15.6)	12 (25.0)	10 (10.8)	
IIIB	6 (4.3)	4 (8.3)	2 (2.2)	

PP, postoperative; IQR, interquartile range; VATS, video-assisted thoracoscopic surgery; RATS, robot-assisted thoracoscopic surgery.

Significant values are in bold.

### Efficacy

**Figure 3:**
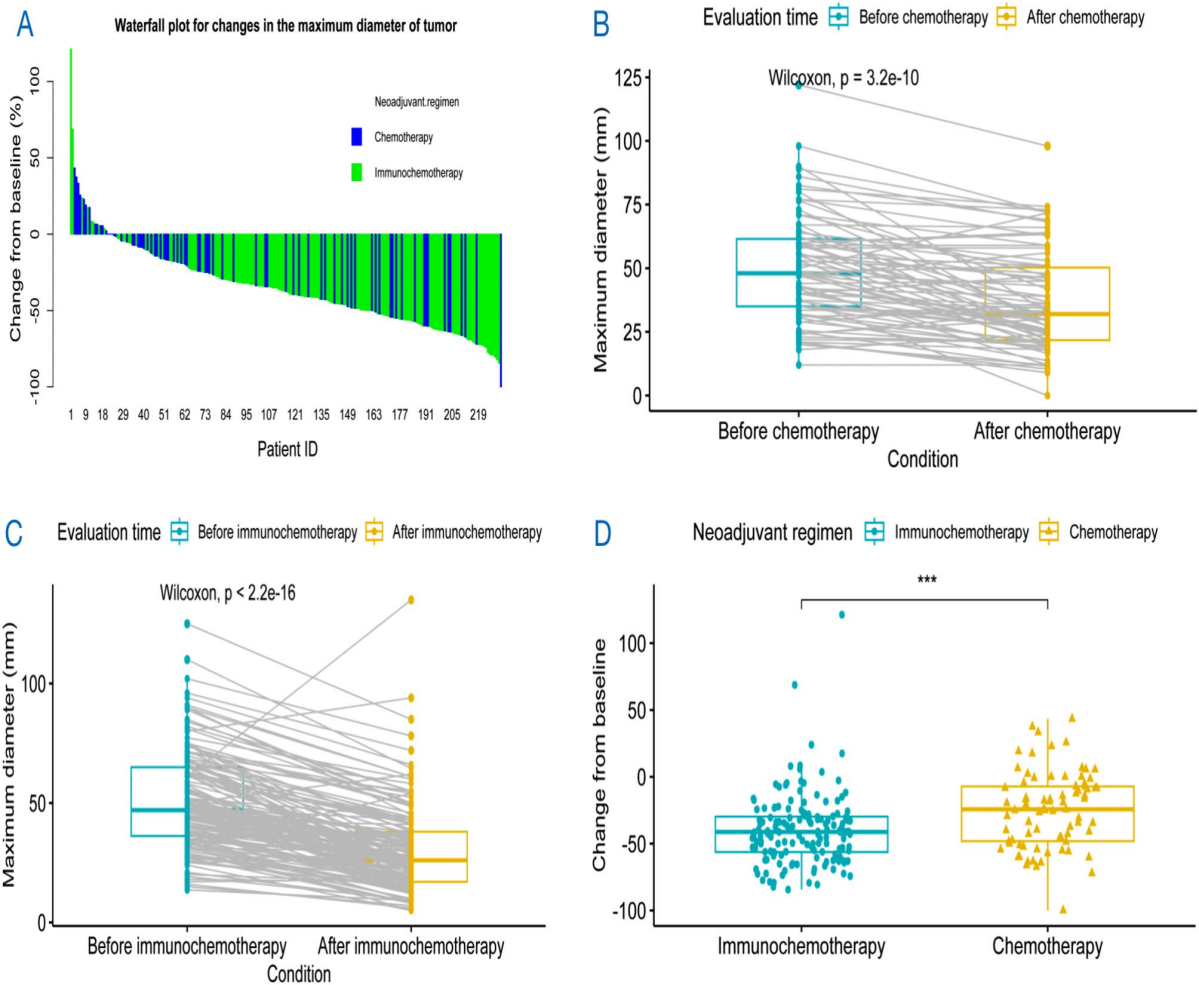
(**A**) The percentage change in the maximum diameter of target lesion compared with the baseline tumor size in the ITT population (n = 231). (**B**) The change of in the maximum diameter of target lesion before and after neoadjuvant chemotherapy. (**C**) The change of in the maximum diameter of target lesion before and after neoadjuvant immunochemotherapy. (**D**) The change of the maximum diameter of target lesion between immunochemotherapy group and chemotherapy group. ITT, intention-to-treat. ***Represents 0.0001 < P value ≤ 0.001.

As shown in Fig. 3A, the maximum diameter of the target lesion of most patients in both groups decreased compared with the baseline tumor size. And significant differences were found in the change of the maximum diameter of target lesion before and after neoadjuvant treatment (Fig. 3B: chemotherapy, Fig. 3C: immunochemotherapy). After neoadjuvant treatment, there was a significant decrease in the number of patients classified as T4, T3, and T2, while the number of patients categorized as T0 and T1 significantly increased (p < 0.05), as indicated in Table 3. Although there was a decrease in the number of patients with N2 and an increase in patients with N0 and N1, this difference was not statistically significant (p > 0.05). Furthermore, the patient population in stage IIIB notably decreased, whereas the numbers in stages I, II, and IIIA saw a significant increase (p < 0.05). What’s more, we observed a significant difference in the change of the maximum diameter of target lesion between two groups (P ≤ 0.001) (Fig. 3D).

**Table 3:** Changes of clinical stage of the ITT population (n = 231) before (cStage) and after (ycStage) neoadjuvant treatment.

Characteristic	Chemotherapy			Immunochemotherapy		
	cStage,	ycStage,	*P*-value	cStage,	ycStage,	*P*-value
	n = 78	n = 78		n = 153	n = 153	
T stage, n (%)			**0.009**			** < 0.001**
T0	0 (0.0)	1 (1.3)		0 (0.0)	0 (0.0)	
T1a	0 (0.0)	1 (1.3)		0 (0.0)	13 (8.5)	
T1b	3 (3.8)	13 (16.7)		6 (3.9)	38 (24.8)	
T1c	11 (14.1)	23 (29.5)		15 (9.8)	42 (27.5)	
T2a	14 (17.9)	12 (15.4)		31 (20.3)	23 (15.0)	
T2b	14 (17.9)	8 (10.3)		35 (22.9)	19 (12.4)	
T3	21 (26.9)	12 (15.4)		36 (23.5)	13 (8.5)	
T4	15 (19.2)	8 (10.3)		30 (19.6)	5 (3.3)	
N stage, n (%)			0.856			0.563
N0	3 (3.8)	5 (6.4)		8 (5.2)	11 (7.2)	
N1	12 (15.4)	13 (16.7)		26 (17.0)	33 (21.6)	
N2	60 (76.9)	58 (74.4)		117 (76.5)	106 (69.3)	
N3	3 (3.8)	2 (2.6)		2 (1.3)	3 (2.0)	
Stage, n (%)			**0.046**			** < 0.001**
0	0 (0.0)	1 (1.3)		0 (0.0)	0 (0.0)	
IA	0 (0.0)	4 (5.1)		0 (0.0)	5 (3.3)	
IB	1 (1.3)	0 (0.0)		4 (2.6)	3 (2.0)	
IC	0 (0.0)	0 (0.0)		0 (0.0)	0 (0.0)	
IIA	1 (1.3)	0 (0.0)		2 (1.3)	2 (1.3)	
IIB	9 (11.5)	11 (14.1)		17 (11.1)	33 (21.6)	
IIIA	32 (41.0)	42 (53.8)		75 (49.0)	91 (59.5)	
IIIB	35 (44.9)	19 (24.4)		55 (35.9)	19 (12.4)	
IVA	0 (0.0)	1 (1.3)		0 (0.0)	0 (0.0)	

ITT, intention-to-treat.

Significant values are in bold.

According to the RECIST 1.1 criteria, most cases in the chemotherapy group presented with a SD, but most patients in the immunochemotherapy group presented with a PR (Table 4). The objective response rate (ORR) of immunochemotherapy regimen was higher than that of the chemotherapy regimen (immunochemotherapy: 74.5%, chemotherapy: 42.3%), and we could see a significant difference (P < 0.001). In terms of the pathological response, the rates of MPR and pCR in the immunochemotherapy group were 66.7% and 41.9%, respectively, which were both higher than that in the other group (MPR: 25.0%, pCR: 20.8%), and a significant difference was observed (P < 0.001).

**Table 4:** Assessments of tumor response to treatment.

	ITT population (n = 231)				PP population (n = 141)		
	Chemotherapy, n = 78	Immunochemotherapy, n = 153	*P*-value		Chemotherapy, n = 48	Immunochemotherapy, n = 93	*P*-value
Therapeutic evaluation, n (%)			** < 0.001**	Pathological response, n (%)			** < 0.001**
CR	2 (2.6)	4 (2.6)		TRG 0	10 (20.8)	39 (41.9)	
PR	31 (39.7)	110 (71.9)		TRG 1	2 (4.2)	23 (24.7)	
SD	40 (51.3)	37 (24.2)		TRG 2	36 (75.0)	31 (33.3)	
PD	5 (6.4)	2 (1.3)					

Abbreviations: ITT, intention-to-treat; PP, postoperative; CR, complete remission; PR, partial remission; SD, stable disease; PD, progression disease; TRG, tumor regression grade.

Significant values are in bold.

**Figure 4:**
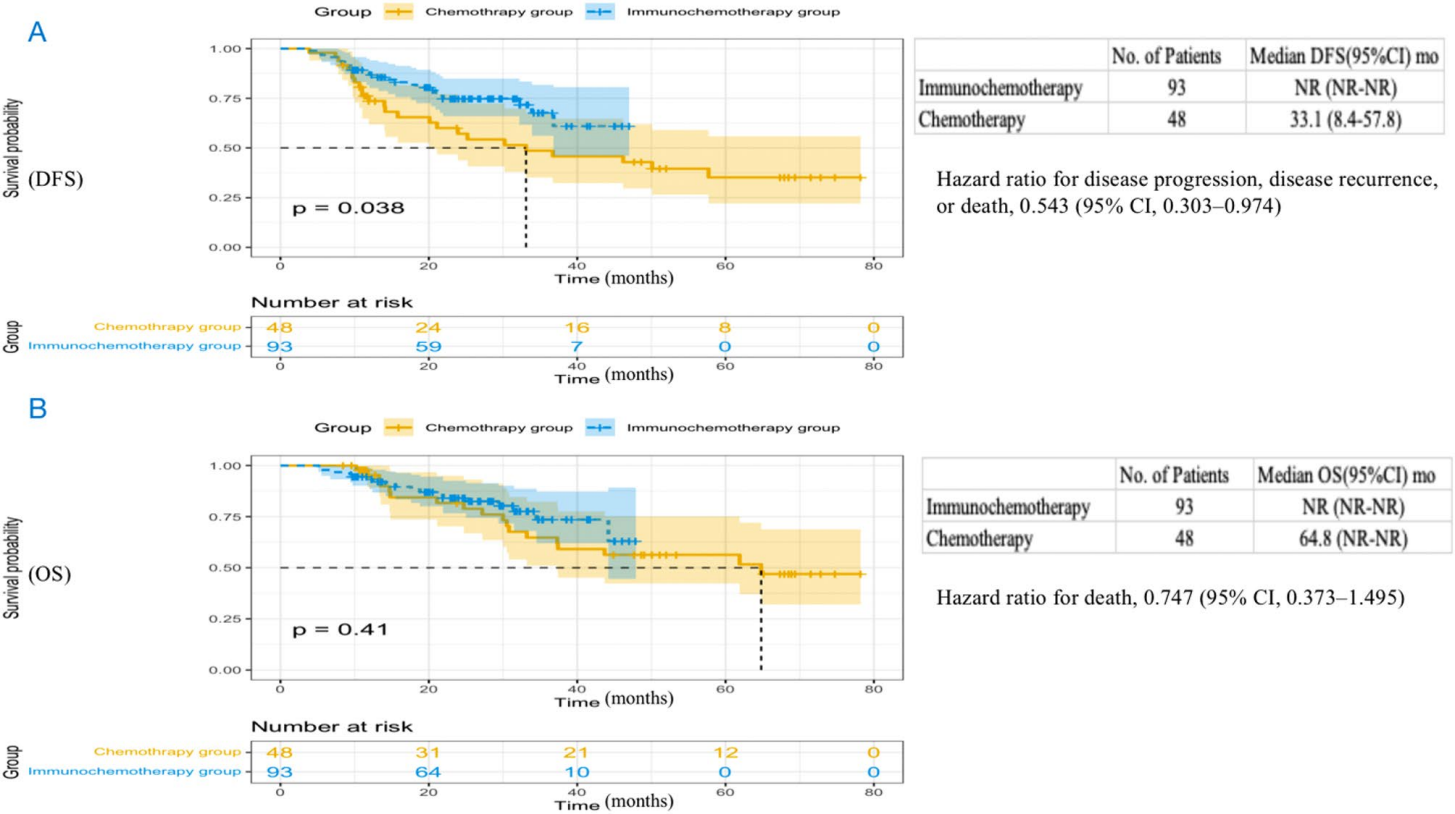
Kaplan Meier curves of disease-free survival (**A**) and overall survival (**B**) in the PP population (n = 141) between the immunochemotherapy group and the chemotherapy group. DFS, disease-free survival; OS, overall survival; CI, confidence interval; NR, not reached.

**Figure 5:**
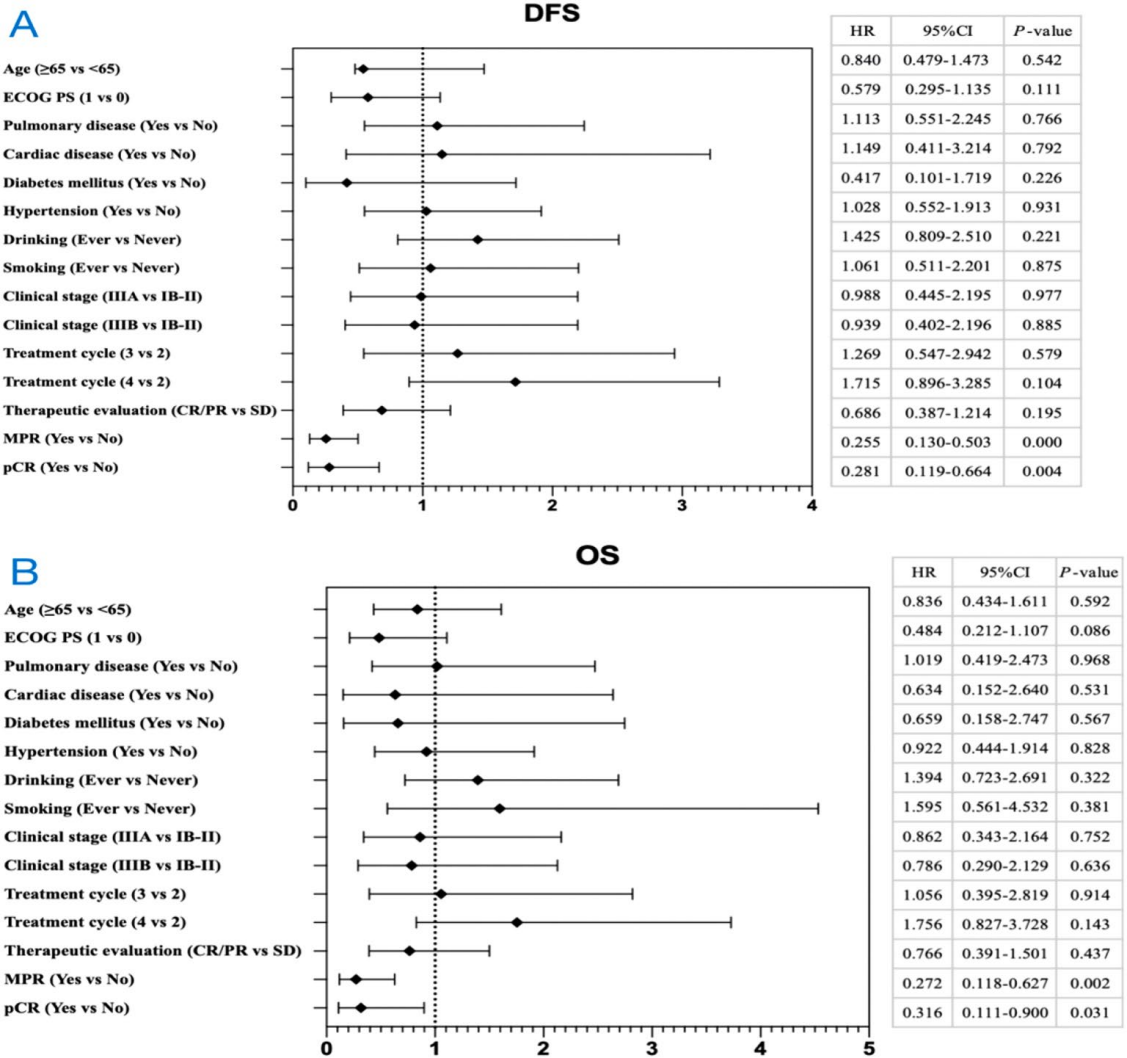
Forest plot of hazard ratio for disease-free survival (**A**) and overall survival (**B**) in the PP population (n = 141). PP, postoperative; DFS, disease-free survival; OS, overall survival; ECOG PS, eastern cooperative oncology group performance status; CR, complete remission; PR, partial remission; SD, stable disease; MPR, major pathologic response; pCR, pathologic complete response; CI, confidence interval; HR, hazard ratio.

At the time of data cutoff (March 2023), we have successfully collected follow-up information in the PP population (93 cases in the immunotherapy group and 48 cases in the chemotherapy group). The median follow-up time for the immunochemotherapy group was 28.7 months (95% confidence interval [CI], 25.9 to 31.5), while the median follow-up time for the chemotherapy group was 52.0 months (95% CI 46.8 to 57.2). Among the immunochemotherapy group, 18 patients experienced recurrence and metastasis, 6 patients died from other causes, and 13 patients died due to recurrence and metastasis. Among the chemotherapy group, 23 patients experienced recurrence and metastasis, 2 patients died from other causes, and 16 patients died due to cancer recurrence and metastasis. The median DFS in the chemotherapy group was 33.1 months (95% CI 8.4 to 57.8) and not reached in the immunochemotherapy group (hazard ratio [HR] for disease progression, disease recurrence, or death, 0.543; 95% CI 0.303 to 0.974; P = 0.038) (Fig. 4A). The 1-year DFS rate, 2-year DFS rate and 3-year DFS rate in the chemotherapy group were 75.0%, 62.5% and 56.3%, with that in the immunochemotherapy group 87.1%, 77.4% and 75.3%. The median OS of the immunochemotherapy group was not achieved (HR for death, 0.747; 95% CI 0.373 to 1.495; P = 0.41), with the chemotherapy group 64.8 months (95% CI not reached to not reached) (Fig. 4B). The 1-year OS rate, 2-year OS rate and 3-year OS rate in the chemotherapy group were 95.8%, 85.4% and 72.9%, with that in the immunochemotherapy group 93.5%, 86.0% and 81.7%. In the context of univariate Cox regression analyses, statistically significant correlations between baseline patient characteristics and DFS (Fig. 5A) or OS (Fig. 5B) were absent, with the exception of MPR (Yes vs No) and pCR (Yes vs No). Notably, individuals with MPR experienced a marked improvement in both DFS and OS, as indicated by hazard ratios (HR) of 0.255 (95% CI 0.130 to 0.503) and 0.272 (95% CI 0.118 to 0.627), respectively. Similarly, patients with pCR exhibited significantly enhanced DFS and OS, with HRs of 0.281 (95% CI 0.119 to 0.664) and 0.316 (95% CI 0.111 to 0.900), respectively.

### Safety

There were no previously undocumented adverse events in our study (Table 5). Overall, the incidence of adverse events (AEs) in the neoadjuvant immunochemotherapy group was 90.8% (139/153) and 80.8% (63/78) in the chemotherapy group. The incidence of leukopenia (53.6% vs 26.9%), anemia (61.4% vs 42.3%), constipation (24.8% vs 11.5%) and skin reaction (26.8% vs 5.1%) in the immunochemotherapy group was significantly higher than that in the chemotherapy group (P < 0.05). There were no significant differences in other adverse reactions between the two groups. The incidence of grade3 and 4 adverse events was 18.3% (28/153) in the immunochemotherapy group and 2.6% (2/78) in the chemotherapy group. Grade 3–4 adverse reactions were mainly distributed in hematological abnormalities. There were no significant differences in the occurrence of grade 3 and grade 4 adverse reactions between the two groups. These adverse reactions were quickly resolved after symptomatic treatment.

**Table 5:** Adverse events of neoadjuvant therapy in the ITT population (n = 231).

Event	Any grade				Grade 3 or 4			
	Total, n = 231	Chemotherapy, n = 78	Immunochemotherapy, n = 153	*P*-value	Total, n = 231	Chemotherapy, n = 78	Immunochemotherapy, n = 153	*P*-value
Hematologic								
Leukopenia	103 (44.6)	21 (26.9)	82 (53.6)	** < 0.001**	7 (3.0)	1 (1.3)	6 (3.9)	0.268
Agranulocytosis	46 (19.9)	13 (16.7)	33 (21.6)	0.378	4 (1.7)	1 (1.3)	3 (2.0)	0.708
Anemia	127 (55.0)	33 (42.3)	94 (61.4)	**0.006**	15 (6.5)	2 (2.6)	13 (8.5)	0.084
Thrombocytopenia	25 (10.8)	6 (7.7)	19 (12.4)	0.274	6 (2.6)	0 (0.0)	6 (3.9)	0.076
Gastrointestinal								
Nausea	6 (2.6)	0 (0.0)	6 (3.9)	0.076	0 (0.0)	0 (0.0)	0 (0.0)	NA
Emesis	14 (6.1)	3 (3.8)	11 (7.2)	0.314	0 (0.0)	0 (0.0)	0 (0.0)	NA
Diarrhea	11 (4.8)	2 (2.6)	9 (5.9)	0.259	1 (0.4)	0 (0.0)	1 (0.7)	0.473
Constipation	47 (20.3)	9 (11.5)	38 (24.8)	**0.018**	2 (0.9)	0 (0.0)	2 (1.3)	0.311
Immune myocarditis	0 (0.0)	0 (0.0)	0 (0.0)	NA	0 (0.0)	0 (0.0)	0 (0.0)	NA
Immune pneumonia	3 (1.3)	0 (0.0)	3 (2.0)	0.213	0 (0.0)	0 (0.0)	0 (0.0)	NA
Hepatic injury	90 (39.0)	35 (44.9)	55 (35.9)	0.188	4 (1.7)	0 (0.0)	4 (2.6)	0.15
Renal injury	11 (4.8)	2 (2.6)	9 (5.9)	0.263	0 (0.0)	0 (0.0)	0 (0.0)	NA
Skin reaction	45 (19.5)	4 (5.1)	41 (26.8)	** < 0.001**	7 (3.0)	0 (0.0)	7 (4.6)	0.055
Hypothyroidism	5 (2.2)	0 (0.0)	5 (3.3)	0.106	0 (0.0)	0 (0.0)	0 (0.0)	NA
Coagulation disorders	3 (1.3)	1 (1.3)	2 (1.3)	0.978	0 (0.0)	0 (0.0)	0 (0.0)	NA
Sensory neurotoxicity	3 (1.3)	1 (1.3)	2 (1.3)	0.978	0 (0.0)	0 (0.0)	0 (0.0)	NA

ITT, intention-to-treat.

Significant values are in bold.

### Survival surrogate analysis

**Figure 6:**
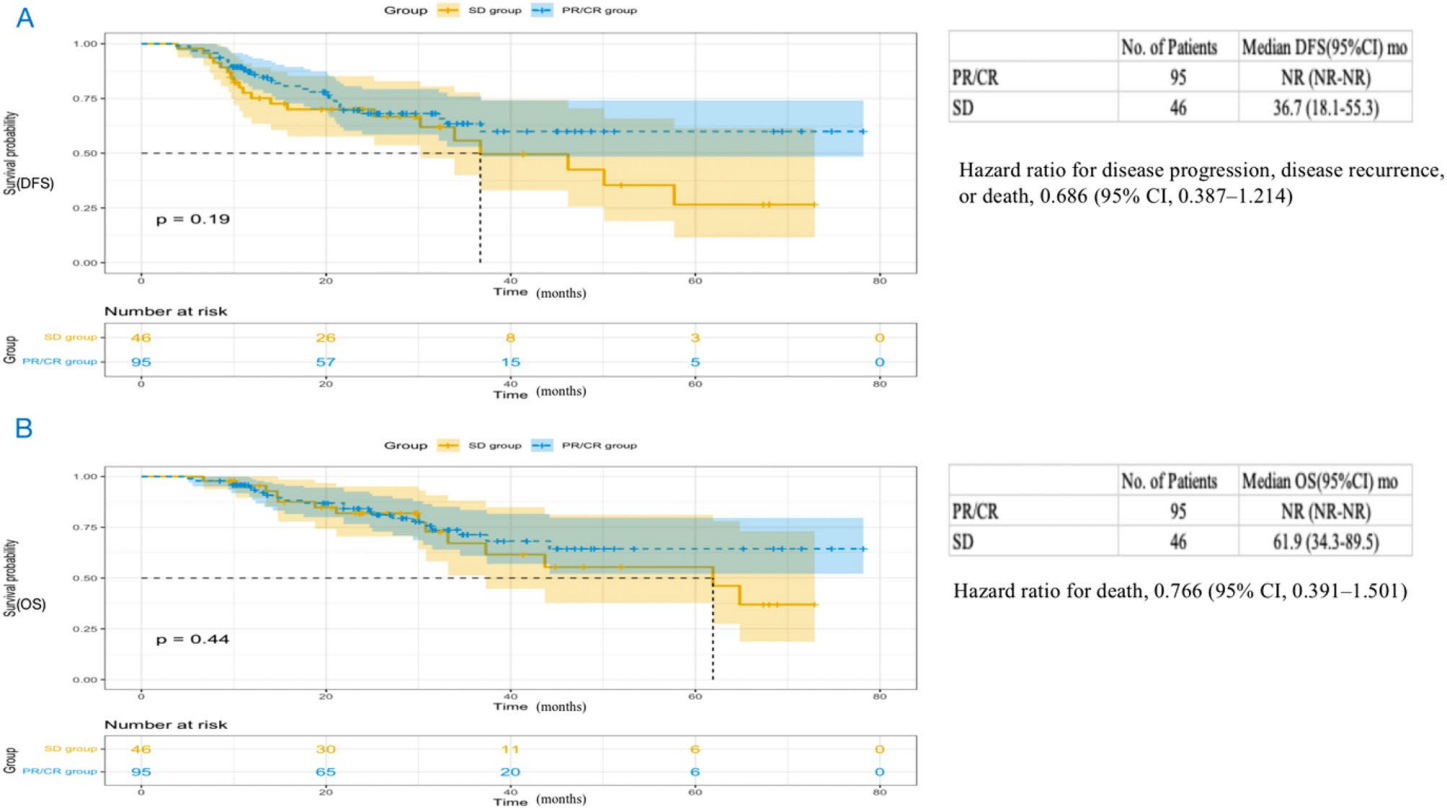
Kaplan Meier curves of disease-free survival (**A**) and overall survival (**B**) in the PP population (n = 141) between the PR/CR group and the SD group. PP, postoperative; DFS, disease-free survival; OS, overall survival; CR, complete remission; PR, partial remission; SD, stable disease; CI, confidence interval; NR, not reached.

**Figure 7:**
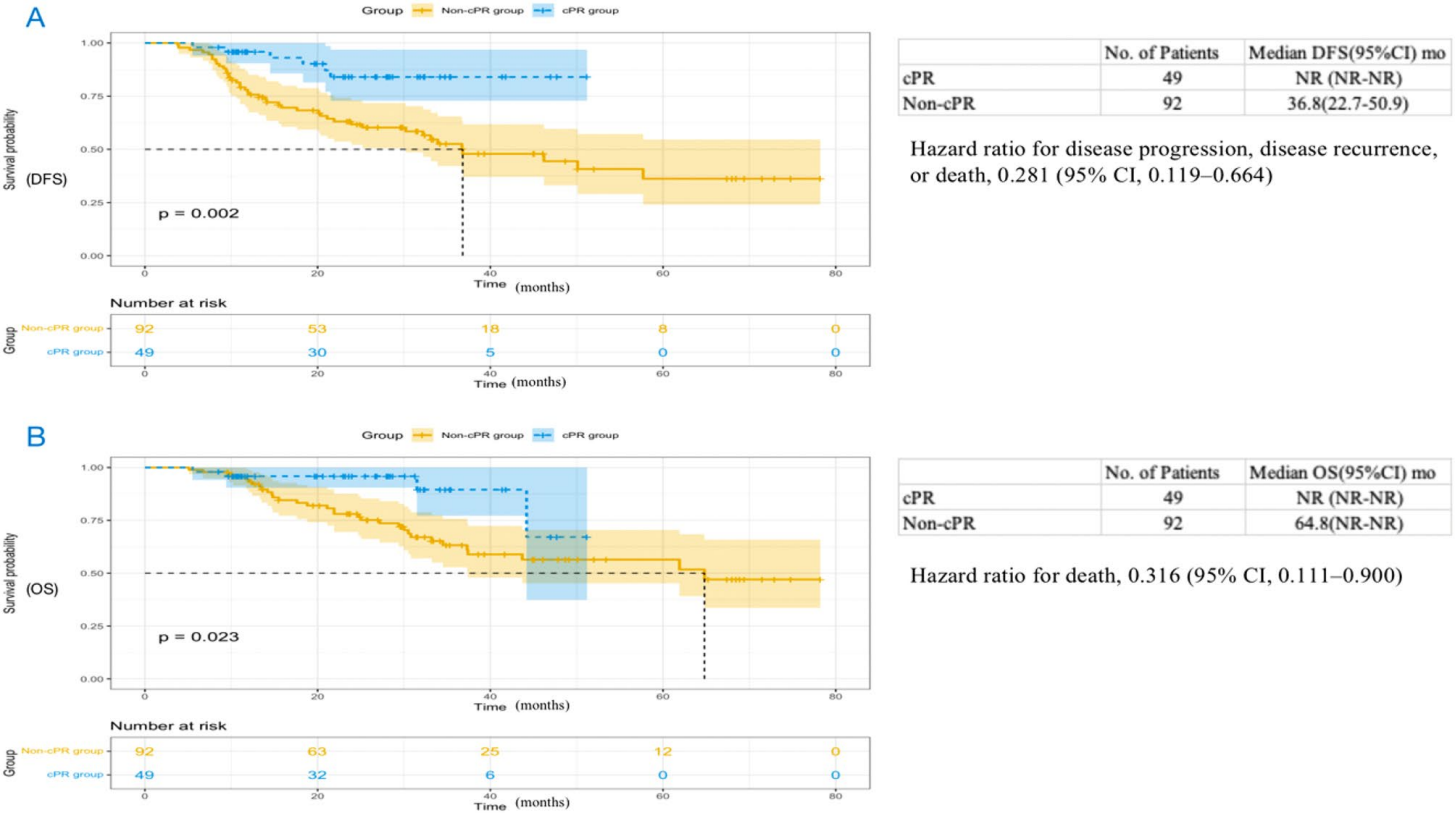
Kaplan Meier curves of disease-free survival (**A**) and overall survival (**B**) in the PP population (n = 141) between the pCR group and the non- pCR group. PP, postoperative; DFS, disease-free survival; OS, overall survival; pCR, pathologic complete response; CI, confidence interval; NR, not reached.

**Figure 8:**
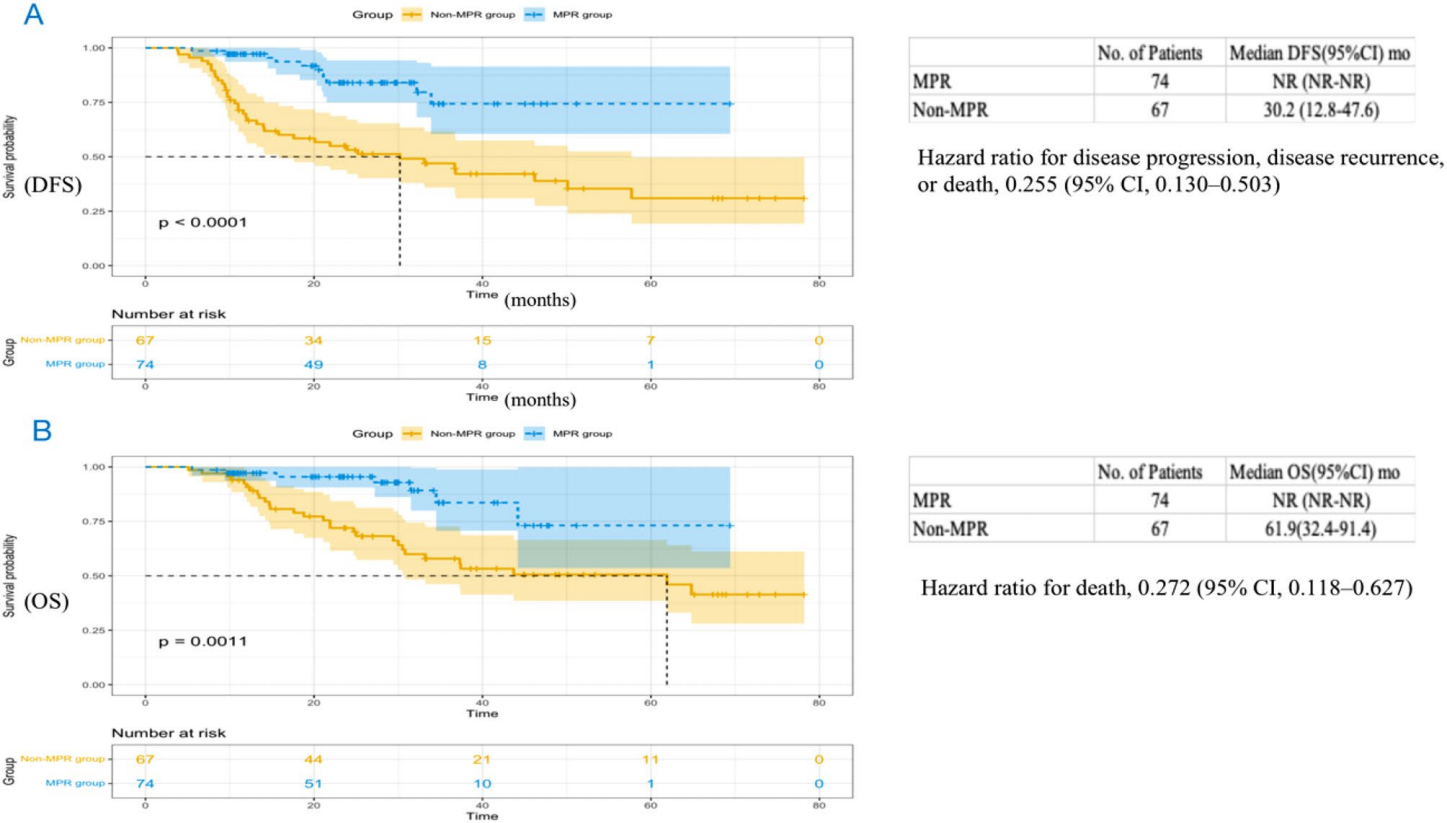
Kaplan Meier curves of disease-free survival (**A**) and overall survival (**B**) in the PP population (n = 141) between the MPR group and the non-MPR group. PP, postoperative; DFS, disease-free survival; OS, overall survival; MPR, major pathologic response; CI, confidence interval; NR, not reached.

As shown in Fig. 6, the median DFS in the SD group was 36.7 months (95% CI 18.1 to 55.3) and not reached in the PR/CR group (HR, 0.686; 95% CI 0.387 to 1.214; P = 0.19), and the median OS of the PR/CR group was also not achieved (HR, 0.747; 95% CI 0.373 to 1.495; P = 0.41), with the SD group 61.9 months (95% CI 34.3 to 89.5). The median DFS in the non-pCR group was 36.8 months (95% CI 22.7 to 50.9) and not reached in the pCR group (HR, 0.281; 95% CI 0.119 to 0.664; P = 0.002) (Fig. 7A). The median OS of the pCR group was not achieved (HR, 0.316; 95% CI 0.111 to 0.900; P = 0.023), with the pCR group 64.8 months (95% CI not reached to not reached) (Fig. 7B). In addition, we found the median DFS in the non-MPR group was 30.2 months (95% CI 12.8 to 47.6) and not reached in the MPR group (HR, 0.255; 95% CI 0.130 to 0.503; P < 0.0001), and the median OS of the MPR group was not achieved (HR for death, 0.272; 95% CI 0.118 to 0.627; P = 0.0011), with the non-MPR group 61.9 months (95% CI 32.4 to 91.4) (Fig. 8).

Multivariate cox regression analyses of survival surrogates (clinical response and pathologic response) were performed in the PP population (n = 141) (Table 6). Improved DFS and OS were not observed in patients with PR/CR (HR, 0.792; 95% CI 0.445 to 1.407; P = 0.426; and HR, 0.906; 95% CI 0.460 to 1.784; P = 0.776 for DFS and OS, respectively). In addition, pCR was not associated with superior DFS and OS (HR, 0.695; 95% CI 0.212 to 2.280; P = 0.548; and HR, 0.849; 95% CI 0.790 to 3.798; P = 0.831, respectively). However, MPR was significantly associated with DFS and OS (HR, 0.325; 95% CI 0.127 to 0.833; P = 0.019; and HR, 0. 906; 95% CI 0.092 to 1.008; P = 0.051, respectively). The C-index of MPR (0.730 for DFS, 0.722 for OS) was higher than the C-index of cPR (0.672 for DFS, 0.659 for OS) and clinical response (0.426 for DFS, 0.542 for OS).

**Table 6:** Multivariate cox regression analyses of survival surrogates (clinical response and pathologic response) in the PP population (n = 141).

Survival surrogate	DFS					OS				
	HR	95%CI	P-value	C-index	95%CI	HR	95%CI	P-value	C-index	95%CI
Clinical response (CR/PR vs SD)	0.792	0.445–1.407	0.426	0.563	0.462–0.664	0.906	0.460–1.784	0.776	0.542	0.431–0.653
Pathologic response (pCR vs non-pCR)	0.695	0.212–2.280	0.548	0.672	0.583–0.762	0.849	0.790–3.798	0.831	0.659	0.563–0.754
Pathologic response (MPR vs non-MPR)	0.325	0.127–0.833	0.019	0.730	0.642–0.818	0.906	0.092–1.008	0.051	0.722	0.628–0.816

PP, postoperative; DFS, disease-free survival; OS, overall survival; HR, hazard ratio; CI, confidence interval; CR, complete remission; PR, partial remission; SD, stable disease; MPR, major pathologic response; pCR, pathologic complete response.

In order to investigate which factors were associated with the MPR, we divided the PP population into MPR group and non-MPR group. Outcomes of univariate analyses were listed in Table 7. There were no significant differences in age, sex, ECOG performance status, smoking status, drinking status, comorbidities, tumor location, T stage, N stage, pathological grade, treatment cycle, and therapeutic evaluation (P > 0.05). There were significant differences in clinical stage (P = 0.048) and therapeutic regimen (P < 0.001). To further verify whether the clinical stage and therapeutic regimen were factors affecting MPR, we included statistically significant variables (clinical stage and therapeutic regimen) in univariate analyses and variable with P < 0.01 (therapeutic evaluation) in the binary logistic regression model for multivariate analysis. Results showed that therapeutic regimen (P < 0.001; OR = 7.406; 95% CI 3.054 to 17.960) was significantly correlated with MPR (Table 8). It can be seen that therapeutic regimen is an independent influencing factor of MPR.

**Table 7:** Outcomes of univariate analyses in the PP population (n = 141) between the MPR group and the non-MPR group.

Characteristic	Total, n = 141	Non-MPR, n = 67	MPR, n = 74	*P*-value
Median age (IQR), years	64.5 (59.0–70.0)	64.0 (58.0–69.0)	65.0 (59.8–71.0)	0.989
Sex, n (%)				0.498
Male	2 (1.4)	0 (0.0)	2 (2.7)	
Female	139 (98.6)	67 (100.0)	72 (97.3)	
ECOG performance status, n (%)				0.582
0	100 (70.9)	49 (73.1)	51 (68.9)	
1	41 (29.1)	18 (26.9)	23 (31.1)	
Smoking status, n (%)				0.213
Never	34 (24.1)	13 (19.4)	21 (28.4)	
Ever	107 (75.9)	54 (80.6)	53 (71.6)	
Drinking status, n (%)				0.672
Never	93 (66.0)	43 (64.2)	50 (67.6)	
Ever	48 (34.0)	24 (35.8)	24 (32.4)	
Comorbidities, n (%)				
Pulmonary disease	28 (19.9)	12 (17.9)	16 (21.6)	0.581
Cardiac disease	11 (7.8)	6 (7.0)	5 (6.8)	0.627
Diabetes mellitus	12 (10.0)	6 (7.0)	6 (8.1)	0.857
Hypertension	39 (27.7)	18 (26.9)	21 (28.4)	0.841
Tumor location, n (%)				0.622
Mediastinum	1 (0.7)	0 (0.0)	1 (1.4)	
Hilum of left lung	3 (2.1)	1 (1.5)	2 (2.7)	
Inferior lobe of left lung	23 (16.3)	10 (14.9)	13 (17.6)	
Inferior lobe of right lung	32 (22.7)	16 (23.9)	16 (21.6)	
Middle lobe of right lung	8 (5.7)	6 (9.0)	2 (2.7)	
Superior lobe of left lung	35 (24.8)	18 (26.9)	17 (23.0)	
Superior lobe of right lung	39 (27.7)	16 (23.9)	23 (31.1)	
T stage, n (%)				0.586
T1b	5 (3.5)	1 (1.5)	4 (5.4)	
T1c	15 (10.6)	8 (11.9)	7 (9.5)	
T2a	24 (17.0)	10 (14.9)	14 (18.9)	
T2b	39 (27.7)	20 (30.0)	19 (25.7)	
T3	36 (25.5)	15 (22.4)	21 (28.4)	
T4	22 (15.6)	13 (19.4)	9 (12.2)	
N stage, n (%)				0.350
N0	6 (4.3)	1 (1.5)	5 (6.8)	
N1	32 (22.7)	16 (23.9)	16 (21.6)	
N2	99 (70.2)	46 (68.7)	53 (71.6)	
Clinical stage, n (%)				**0.048**
IB	3 (2.1)	0 (0.0)	3 (4.1)	
IIA	2 (1.4)	0 (0.0)	2 (2.7)	
IIB	21 (14.9)	13 (19.4)	8 (10.8)	
IIIA	67 (47.5)	27 (40.3)	40 (54.1)	
IIIB	48 (34.0)	27 (40.3)	21 (28.4)	
Pathological grade, n (%)				0.686
G1	8 (5.7)	4 (6.0)	4 (5.4)	
G2	54 (38.3)	28 (41.8)	26 (35.1)	
G3	79 (56.0)	35 (52.2)	44 (59.5)	
Therapeutic regimen, n (%)				** < 0.001**
Chemotherapy	48 (34.0)	36 (53.7)	12 (16.2)	
Immunochemotherapy	93 (66.0)	31 (46.3)	62 (83.8)	
Treatment cycle, n (%)				0.296
2	73 (51.8)	34 (50.7)	39 (52.7)	
3	17 (12.1)	11 (16.4)	6 (8.1)	
4	51 (36.2)	22 (32.8)	29 (39.2)	
Therapeutic evaluation, n (%)				**0.064**
CR/PR	95 (67.4)	40 (59.7)	55 (74.3)	
SD	46 (32.6)	27 (40.3)	19 (25.7)	

PP, postoperative; MPR, major pathologic response; IQR, interquartile range; ECOG, Eastern Cooperative Oncology Group; CR, complete remission; PR, partial remission; SD, stable disease.

Significant values are in bold.

**Table 8:** Outcomes of multivariate analyses in the PP population (n = 141) between the MPR group and the non-MPR group.

Characteristic	*P*-value	OR	95%CI
Clinical stage			
IB	0.999	0.000	–
IIA	0.999	0.000	–
IIB	–	1	–
IIIA	0.083	2.717	0.878–8.409
IIIB	0.939	1.046	0.328–3.333
Therapeutic evaluation			
SD	–	1	–
CR/PR	0.515	1.324	0.568–3.085
Therapeutic regimen			
Chemotherapy	–	1	–
Immunochemotherapy	** < 0.001**	7.406	3.054–17.960

PP, postoperative; OR, odds ratio; CR, complete remission; PR, partial remission; SD, stable disease.

Significant values are in bold.

## Discussion

In recent years, the feasibility and safety of neoadjuvant immunotherapy combined with chemotherapy have been validated in the treatment of stage IB-IIIB NSCLC^18–20^. However, there are still few clinical studies focusing on evaluating the tolerance and efficacy of neoadjuvant immunotherapy combined with chemotherapy in previously untreated stage IB-IIIB LUSC. Our study found that neoadjuvant immunochemotherapy resulted in better survival benefit (median DFS: not reached vs 33.1 months, P = 0.038, HR, 0.543; median OS: not reached vs 64.8 months, P = 0.41, HR, 0.747) than chemotherapy among patients with stage IB to IIIB LUSC. The 2-year DFS rate and 2-year OS rate in the immunochemotherapy group were 77.4% and 86.0%, with that in the chemotherapy group 62.5% and 85.4%. Survival results of this study were superior than those of NADIM II study^10^. It showed that the median progression-free survival (PFS) in the chemotherapy group was not reached and 15.4 months in the chemotherapy group (HR, 0.47), and the median OS was not reached in both groups (HR, 0.43). The 2-year PFS rate and 2-year OS rate in the immunochemotherapy group were 67.2% and 85.0%, with that in the chemotherapy group 40.9% and 63.6%. Our study also found that neoadjuvant immunochemotherapy produced a higher percentage of patients with a MPR (including pCR) than chemotherapy. In our study, MPR rates were 66.7% for the immunochemotherapy group and 25.0% for the chemotherapy group, while pCR rates stood at 41.9% for the former and 20.8% for the latter (p < 0.001). Notably, these findings exhibited superior outcomes compared to those observed in the NADIM II study, where the immunochemotherapy group achieved a pCR rate of 37%, whereas the chemotherapy group only reached 7% (p = 0.02)^10^. In the Checkmate-816 study, the rate of pCR in the immunochemotherapy group and the chemotherapy group was 24.0% and 2.2%, respectively (P < 0.001)^9^. The variations observed in these studies could potentially stem from variations in drug regimens employed, treatment cycle disparities, variations in the incidence of lung squamous cell carcinoma, and differences in the staging criteria applied to the study populations.

Neoadjuvant treatment can reduce tumor size and even achieve a downgrading effect, allowing patients who cannot undergo surgery before neoadjuvant therapy to complete surgery after treatment, achieving more thorough tumor resection and achieving better results. Stage IIIB non-small cell lung cancer is traditionally classified as unresectable; however, the introduction of neoadjuvant therapy has revolutionized this outlook. As evidenced by our study, following neoadjuvant treatment, a notable reduction was observed in the number of patients with T4, T3, T2, and N2, resulting in an increase in patients with T0, T1, N0, N1, stage I, stage II, and stage IIIA. Ultimately, surgical intervention was performed in 60.8% of patients in the immunochemotherapy group and 61.5% in the chemotherapy group. But this was lower than that in the Checkmate-816 study (83.2% vs 75.4%) and the NADIM II study (93% vs 69%)^9,10^. The reason for the lower surgical resection rate in our immunochemotherapy group might be that a higher percentage of patients in our immunochemotherapy group were reluctant to undergo surgery. Open surgery was performed in most of patients of these two groups (immunochemotherapy group:62.4%, chemotherapy group:70.8%), which was higher than the Checkmate-816 study (59.2% vs 63.0%)^9^. The reason for the higher percentage of open surgery in our study might be the existence of patients with stage IIIB. The percentage of patients who underwent pneumonectomy was similar in in these two groups (10.8% in the immunochemotherapy group and 10.4% in the chemotherapy group), which was similar to the NADIM II study (10% vs 11%) and lower than the Checkmate-816 study (16.8% vs 25.2%)^9,10^. The rate of R0 resection in the immunochemotherapy group was higher than that in the chemotherapy group (94.6% vs 77.8%), which was similar to the NADIM II study (94% vs 85%) and lower than the Checkmate-816 study (83.2% vs 77.8%)^9,10^. Adjuvant treatment (such as: immunochemotherapy, immunotherapy, chemotherapy or radiotherapy) was received by 90.3% of the patients in the immunochemotherapy group and 89.6% of those in the chemotherapy group, which was similar to the NADIM II study and different from the Checkmate-816 study^9,10^.

In this study, all of treatment-related AEs were manageable and tolerable. And no new or unexpected AEs were observed. The incidence of AEs in the immunochemotherapy group and the chemotherapy group was 90.8% and 80.8%. The incidence of grade3 and 4 AEs was 18.3% in the immunochemotherapy group and 2.6% in the chemotherapy group. The incidence of AEs related to immunotherapy varied in different clinical researches. In the Checkmate-816 study, the incidence of AEs in the immunochemotherapy group and the chemotherapy group was 92.6% and 97.2%^9^. And the incidence of grade3 and 4 AEs was 40.9% in the immunochemotherapy group and 43.8% in the chemotherapy group. In the NADIM II study, the incidence of AEs in the immunochemotherapy group and the chemotherapy group was 88% and 90%^10^. And the incidence of grade3 and 4 AEs was 19% in the immunochemotherapy group and 10% in the chemotherapy group. The variation in adverse event incidence across these studies might arise from variances in treatment cycle numbers and the utilization of immunotherapy medications.

Although OS is still the gold indicator for evaluating efficacy of neoadjuvant therapy, the process is too long, labor-intensive. Therefore, many alternative indicators for OS have gradually emerged in clinical practice. Increasing numbers of individuals endorse the notion that pathological responses (MPR or pCR) observed in resected specimens can function as surrogate endpoints for survival, offering a more precise and expeditious means of comparing various neoadjuvant treatment regimens. This approach reduces the duration needed to evaluate novel chemotherapeutic and biological therapies in clinical trials^12,21,22^. Studies have shown pathological response was associated with longer survival benefit in NSCLC^9,10,13,14^. In our study, we performed analyses of survival surrogates (clinical response and pathologic response) and found patients with MPR and pCR had significantly improved DFS and OS in univariate Cox regression analyses. After multivariate cox regression analyses, pCR was not associated with superior DFS and OS, but MPR was significantly associated with DFS and OS. In addition, Harrell’s concordance index (C-index) was calculated to evaluate the ability of each survival surrogate to distinguish between dead and non-dead patients, as well as between progressing and non-progressing patients. The C-index of MPR (0.730 for DFS, 0.722 for OS) was higher than the C-index of cPR (0.672 for DFS, 0.659 for OS) and clinical response (0.426 for DFS, 0.542 for OS). All of these results prompted us to postulate that MPR may serve as a surrogate endpoint of survival to evaluate neoadjuvant therapy. We also investigated which factors were associated with the MPR. Results showed that therapeutic regimen (P < 0.001; OR = 7.406; 95% CI 3.054 to 17.960) was significantly correlated with MPR. It can be seen that therapeutic regimen is an independent influencing factor of MPR. Neoadjuvant immunotherapy combined with chemotherapy may bring better MPR.

There are some limitations in this study. First, our study was a retrospective clinical trial and the sample size was small. Second, there was heterogeneity in the selected patients and treatment regimens in this study, which may have some impacts on the results. Therefore, randomized controlled trials and prospective trials with larger scales are required to further validate our outcomes.

In conclusion, neoadjuvant immunochemotherapy can produce a higher percentage of patients with a MPR and longer survival than chemotherapy alone in patients with stage IB to IIIB LUSC. And MPR may serve as a surrogate endpoint of survival to evaluate neoadjuvant therapy.

## Data Availability

The data of the current study are available from the corresponding author on reasonable request.
